# Intensive Care Management of an Adult Patient With Rare Chilaiditi Syndrome Presenting as Abdominal Breathing: A Case Report

**DOI:** 10.7759/cureus.53615

**Published:** 2024-02-05

**Authors:** Gulseren Yilmaz, Kubra Mentese, Pelin Kilic Erol, Bedih Balkan, Ummihan Topal

**Affiliations:** 1 Anesthesiology & Reanimation, Istanbul Kanuni Sultan Suleyman Training and Research Hospital, Istanbul, TUR; 2 Anesthesiology & Reanimation, Basaksehir Cam and Sakura City Training and Research Hospital, Istanbul, TUR; 3 Adult Intensive Care, Istanbul Kanuni Sultan Suleyman Training and Research Hospital, Istanbul, TUR; 4 Interventional Radiology, Istanbul Kanuni Sultan Suleyman Training and Research Hospital, Istanbul, TUR

**Keywords:** radiology, respiratory physiotherapy, icu management, abdominal pain, chilaiditi syndrome

## Abstract

Chilaiditi syndrome (CS) is an uncommon case of the asymptomatic radiographic finding of an intestinal loop between the liver and the diaphragm. The most crucial phases in the diagnosis process are a thorough physical examination and precise imaging, particularly in challenging disorders such as CS. The presence of free air under the right hemidiaphragm in this syndrome, the diagnosis of which is based on radiographic imaging, might direct the start of treatment without the need for surgical intervention. An 86-year-old man, with asthma and chronic obstructive pulmonary disease (COPD) was checked out in our hospital's emergency department (ED) after experiencing nausea and vomiting. Having abdominal breathing while the patient was in an internal medicine department owing to a urinary tract infection (UTI) and acute kidney injury (AKI), he was moved to the intensive care unit (ICU). The patient was treated with respiratory physiotherapy, inhaler bronchodilator treatment, antibiotic therapy, enema, and laxatives. Medical imaging is the primary diagnostic tool for CS, guided by the symptoms. In patients like this elderly patient who was taken to ICU from internal medicine due to acute respiratory failure and abdominal breathing, when free air is detected in the subdiaphragmatic region, control should be provided with computed tomography (CT), and non-invasive mechanical ventilation should be applied.

## Introduction

Interstitial colon was first reported by Greek scholar Demetrius Chilaiditi in 1910, also known as Chilaiditi syndrome (CS), which refers to the colon (mostly hepatic flexure) entering between the liver and diaphragm from the anterior hepatic hiatus or posterior hepatic hiatus, and the cause of which does not lie in the colon itself, but in the downward displacement of the liver and the positional anomalies that are the basis for its formation [1]. CS has a prevalence of 0.025%-0.28%, with a male-to-female ratio of about 4:1, and is more common in the middle-aged and elderly, as well as in those with mental retardation. This disease is usually asymptomatic, mostly discovered by chance during chest or abdominal radiographs, and some patients present with vague pain in the quarter ribs, anorexia, abdominal distension, constipation. When obstruction or torsion occurs in the embedded colon, there may be sudden severe epigastric pain, nausea and vomiting, and respiratory effort, which disappears after a few hours to a few days and is most often relieved suddenly after activity. Although some patients with CS are treated with surgery, conservative medical treatment remains the best treatment option, as symptoms often resolve on their own [2-7].

Variations in the anatomical structure can result in the pathological occurrence of colon interposition observed in CS. These variations may involve the absence, looseness, or elongation of the suspensory ligaments of the transverse colon or falciform ligament. Factors contributing to the predisposition to CS encompass congenital malpositions, functional issues such as persistent constipation arising from elongation and redundancy of the colon, colonic distension with gas, diminished liver size due to cirrhosis or hepatectomy, ascites stemming from elevated intraabdominal pressure, significant weight loss in obese individuals, and anomalies such as an unusually high diaphragm or diaphragmatic paralysis (manifesting in conditions such as diaphragmatic muscular degeneration or phrenic nerve injury). Furthermore, chronic obstructive lung disease, leading to enlargement of the lower thoracic cavity, and multiple pregnancies are additional factors associated with the development of CS [8].

X-ray imaging of the chest or abdomen can show the situation that is temporal or permanent interposition of the segments of the colon (generally) and/or small intestine in CS patients [3]. A physical examination and accurate imaging are the most important steps for diagnosis. In the diagnosis of CS, which is based on radiographic imaging, the detection of free air under the right hemidiaphragm can guide the beginning of treatment without the need for surgical intervention [6]. Herein, we present a patient who was previously diagnosed with asthma and chronic obstructive pulmonary disease (COPD) and performed abdominal breathing after nausea and vomiting complaints. In the posteroanterior chest X-ray, the right hemidiaphragm was elevated more than normal, and colonic loops were observed between the liver and the liver under the diaphragm. Colon interposition between the liver and the diaphragm was also seen on abdominal computed tomography (CT). A case of CS was suspected.

The initial investigation is a plain radiograph, which reveals distinctive air below the diaphragm with apparent colonic haustra that does not appear to follow a postural pattern. When abdominal symptoms and indications are present, a cross-sectional imaging test, such as a CT scan, is necessary to rule out perforated bowel; this is especially true when an ultrasound and plain radiograph are ambiguous. Due to the shortcomings in the patient's history and the challenges in interpreting physical symptoms, CT can be a helpful non-invasive modality in patients with stomach pain to confirm CS and prevent missing a dangerous pathology [7].

## Case presentation

An 86-year-old man, previously diagnosed with asthma and COPD, was admitted to the emergency department (ED) due to acute constipation with nausea and vomiting. The intestines could not perform their normal functions since they were displaced under the diaphragm. An intestinal obstruction and obstructed hernias were ruled out by the absence of abdominal distention during the abdominal examination. He was transferred to the intensive care unit (ICU) after he had abdominal breathing while he was an inpatient in internal medicine service due to a urinary tract infection (UTI) and acute kidney injury (AKI). On physical examination, the vital signs were as follows: blood pressure of 118/85 mmHg and a heart rate of 92 beats per min. Additionally, coarsened rhonchi, wheezing, and abdominal breathing were observed.

Upon the development of respiratory distress in the service follow-ups, the patient who was consulted for the indication of intensive care follow-up was transferred to our ICU due to respiratory failure. The hemogram result was normal. The biochemistry findings are presented in Table 1. Staphylococcus hominis growth was detected in a vial from the blood culture taken.

**Table 1:** Biochemical findings of the case report. CRP: C-reactive protein

Parameter	Findings	Reference Range
Creatinine	1.6 mg/dL	0.50-0.90
Urea	78 mg/dL	17-43
CRP (turbidimetric)	87 mg/L	<5
pH in blood gas	7.27	-
pCO_2_	55 mmHg	35-45
HCO_3_	21 mmol/L	21-29

**Figure 1:**
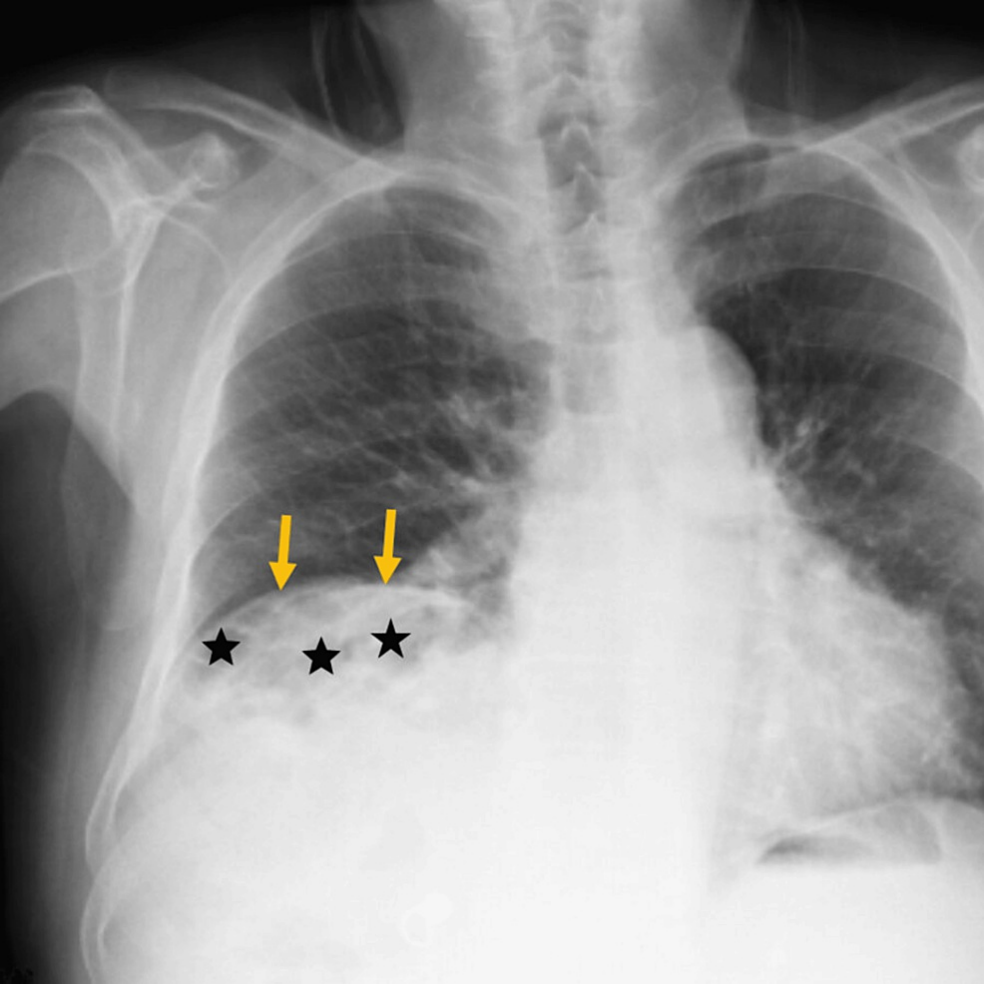
Chest radiograph shows elevation of the right hemidiaphragm (yellow arrows). Radiolucent views (asterisk) representing the interposition of bowel loops are observed.

In the posteroanterior chest X-ray, the right hemidiaphragm was elevated more than normal (Figure 1).

**Figure 2:**
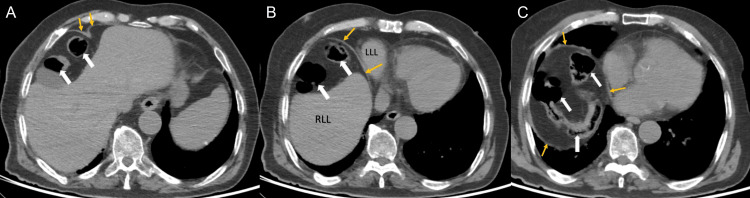
Abdominal intravenous contrast-enhanced computed tomography scans show a loop of bowel inserted between the liver and the hemidiaphragm (yellow arrows) in axial view (white arrows). RLL: right liver lobe, LLL: left liver lobe (A, B, and C).

The colonic loops were observed between the liver and the liver under the diaphragm. Colon interposition was observed between the liver and diaphragm in CT of the abdomen (Figure 2).

When these findings and the patient's history were combined, CS was decided as the diagnosis. In line with the patient's complaints and blood findings including erythrocytopenia and thrombocytopenia, the pooled thrombocytes and erythrocytes were injected in the patient. As a post-diagnosis treatment, the patient was treated with noninvasive mechanical ventilation in the ICU.

The patient was followed up in the ICU for 13 days with respiratory physiotherapy, inhaler bronchodilator therapy, antibiotherapy, enema, and laxative therapy. He was transferred to the internal medicine service after his breathing improved. He was discharged home after being followed up for one day in the service.

## Discussion

The Chilaiditi sign is the hallmark of CS and is typically discovered by accident during a CT scan of the abdomen or chest. According to reports in the literature, CS demonstrated with abdomen CT in between 1.18% and 2.40% of cases. The right hemidiaphragm must be elevated above the liver by the intestine to indicate the Chilaiditi sign, the bowel must be distended by air to demonstrate pseudo-pneumoperitoneum, and the superior margin of the liver must be depressed below the level of the left hemidiaphragm [6-8].

When this radiographic result is identified, the primary objective should be to determine if the subdiaphragmatic air is free or intraluminal. For instance, haustral folds should be visible, and air should not be impacted by positional changes if air has accumulated in the large intestine. However, a second radiograph with positional alterations should show air movement if air has instead accumulated in the peritoneal cavity. CT can be used to confirm anything in question [4]. Similarly, for the situation we presented in this report, abdominal CT showed that there was a colon interposition between the liver and the diaphragm. After the patient was taken to the intensive care unit for non-invasive mechanical ventilation treatment, he was followed up in the intensive care unit for 13 days with treatment methods such as respiratory physiotherapy, inhaler bronchodilator treatment, antibiotic therapy, enema, and laxatives. His treatment was discontinued after respiratory improvement was observed.

In the case report reported by Ali et al. in 2020, CS was detected after an 81-year-old female patient with a similar profile to our case presented to the ED with chest pain, associated with nausea, diffuse abdominal pain, and constipation. Following additional testing, a CT scan of the abdomen showed that the colon was positioned on the diaphragm dome in accordance with the Chilaiditi sign. Based on the unique radiological result and the patient's clinical presentation, CS was identified as the cause of the condition [9]. Thus, correct preliminary examination and accurate radiological imaging requests have provided the patient with positive treatment results without the need for surgical intervention.

## Conclusions

The primary diagnostic tool for CS, guided by symptoms, is medical imaging. In patients like this elderly patient who was taken to the ICU from internal medicine due to acute respiratory failure and abdominal breathing, when free air is detected in the subdiaphragmatic region, control should be provided with abdominal CT. It should be said that, since the case we present in this report is elderly, the diagnosis and treatment made without the need for surgical intervention positively affected the patient's quality of life after treatment.
